# HTLV-1 bZIP factor induces systemic inflammations in vivo

**DOI:** 10.1186/1742-4690-8-S1-A8

**Published:** 2011-06-06

**Authors:** Nanae Taguchi, Yorifumi Satou, Koichi Ohshima, Masao Matsuoka

**Affiliations:** 1 Institute for Virus Research, Kyoto University, Kyoto, 606-8507, Japan; 2 Department of Pathology, Kurume University School of Medicine, Kurume, 830-0011, Japan

## 

Human T-cell leukemia virus type 1 (HTLV-1) causes both neoplastic and inflammatory diseases, which include HTLV-1 associated myelopathy/tropic spastic paraparesis and uveitis. Recently, we have reported that transgenic expression of HTLV-1 bZIP factor (HBZ) in CD4+ T cells caused dermatitis and alveolitis in mice. In this study, we investigated the production of cytokines in HBZ transgenic (HBZ-Tg) mice to elucidate the mechanism of its pro-inflammatory phenotype. IFN-γ production in CD4+ T cells was remarkably increased in splenocytes, lungs and PBMCs from HBZ-Tg compared with non-transgenic littermates, whereas there was no difference in levels of IL-4 and IL-17. We also found that production of IFN-γ was remarkably enhanced in CD4+Foxp3- fraction.

Recent studies have reported that CD4+Foxp3+ T cells are not terminally differentiated but have a plasticity to convert to other T cell subsets. Induced regulatory T cells (iTreg) tend to lose Foxp3 expression, and may acquire an effector phenotype accompanied by the production of inflammatory cytokines, such as IFN-γ. We observed that the percentage of naturally occurring Treg cells was lower in HBZ-Tg mice than non-Tg mice, although total number of Treg was increased in HBZ-Tg mice. It is suggested that the enhanced generation of iTreg cells and instability of Foxp3 expression in HBZ-induced iTreg is a possible mechanism for increased number IFN-γ producing cells in HBZ-Tg mice, leading to systemic inflammation.

